# High Resolution γ-Ray Spectroscopy: the First 85 Years

**DOI:** 10.6028/jres.105.002

**Published:** 2000-02-01

**Authors:** Richard D. Deslattes

**Affiliations:** National Institute of Standards and Technology, Gaithersburg, MD 20899-0001

**Keywords:** crystal diffraction, gamma-ray spectra, instrumentation, precision measurement

## Abstract

This opening review attempts to follow the main trends in crystal diffraction spectrometry of nuclear γ rays from its 1914 beginning in Rutherford’s laboratory to the ultra-high resolution instrumentation realized in the current generation of spectrometers at the Institute Laue Langeven (ILL). My perspective is that of an instrumentalist hoping to convey a sense of our intellectual debt to a number of predecessors, each of whom realized a certain elegance in making the tools that have enabled much good science, including that to which the remainder of this workshop is dedicated. This overview follows some of the main ideas along a trajectory toward higher resolution at higher energies, thereby enabling not only the disentangling of dense spectra, but also allowing detailed study of aspects of spectral profiles sensitive to excited state lifetimes and inter-atomic potentials. The parallel evolution toward increasing efficiency while preserving needed resolution is also an interesting story of artful compromise that should not be neglected. Finally, it is the robustness of the measurement chain connecting γ-ray wavelengths with optical wavelengths associated with the Rydberg constant that only recently has allowed γ-ray data to contribute to determination of particle masses and fundamental constants, as will be described in more detail in other papers from this workshop.

## 1. Introduction

A majority of the contributions to this workshop were enabled by the capabilities of the high-resolution γ-ray spectrometers at the Institute Laue Langeven (ILL). Among these, one originating at the National Institute of Standards and Technology (NIST) and operating as a NIST-ILL collaboration has made particular contributions to the evaluation of picosecond to femtosecond nuclear lifetimes, the determination of inter-atomic potential functions, and accurate measurement of γ-ray transition energies. This introductory contribution attempts to trace the historical evolution of γ-ray diffraction instruments. Such an historical perspective offers a useful structure within which to appreciate some of the key ideas. The story begins with Rutherford and Andrade in 1914 [1,2], it continues with the curved crystal geometry developed by Cauchois (1932–34) [3–5], a configuration that was later adapted to γ-ray spectroscopy [6]. Later there was the large-radius instrument of DuMond in 1947 [7], and the parallel development continued with the double flat crystal instrument at Chalk River [8], GAMS2, 3 at the ILL [9], GAMS4 at the ILL [10], and finally to GAMS5, as is described elsewhere in these proceedings [11]. This coarse-grained sampling neglects a number of important antecedents, such as the double flat crystal instrument of Cuykendall and Jones in 1935 [12], and other high performance bent crystal instruments [13].

Aside from the granularity of the timeline, one has to appreciate the important contributions to γ-ray spectroscopy from developments in other fields, as well as the spin-off of γ-ray spectrometric techniques to still other application areas. In this connection, it should be noted that only a small part of what will be discussed here was anticipated during our planning and development of GAMS4. Fortunately, what was anticipated has also come to pass, as is reported by Kessler and Dewey elsewhere in these proceedings [14]. Beyond these particulars, one cannot fail to acknowledge the generous foresight of the ILL founders in making long-term strategic investments in an enterprise whose character and success could neither have been anticipated nor guaranteed.

Although it is clear that instrumentation does not beget science, in some cases it does enable new science, as is evident in several contributions to these proceedings. In assembling this brief history, my intent is to suggest that at least part of the good work of tomorrow will be a legacy of prior work, some of whose lessons will have been transferable. In the end, I must address the question of where are the physical, i.e., nontechnical performance limits to spectroscopy in the γ-ray region.

## 2. Foundations of -Ray Spectrometry: 1914–1954

While there has been continuing progress in other forms of γ-ray spectroscopy, for example, photo-electron spectroscopy, conversion electron spectroscopy, as well as the very widely used ionization detector/pulse height analysis approach, the present overview is confined entirely to instrumentation using crystal diffraction. This restriction is based on the potential and already achieved resolution values; window function widths below 10^−5^ are at hand, and 10^−4^ is routinely realized. Also the instrumentation is accessible, not only at the ILL but in other institutions as well. In addition, a sub-class of the diffraction instruments provides the only available measurement chain connecting the γ-ray region with secondary standards in the visible that are, in turn, linked to the definition of frequency, and to fundamental atomic constants, the Rydberg in particular. Finally, this kind of instrumentation has shown itself adaptable to new areas including measurement of excited state nuclear lifetimes, and determination of inter-atomic potential functions, as seen in other contributions to these proceedings. The historical markers referred to above were starting points for each of the several forms of crystal diffraction spectroscopy that have been usefully applied in the γ-ray region. In all cases, the full potential of each approach was only realized at a later time, sometimes a much later one. In the paragraphs that follow, the pioneering configurations are described, followed by a sampling of their second and third generation descendants.

### 2.1 Elementary Aspects of Crystal Diffraction Spectroscopy

**Fig. 1:**
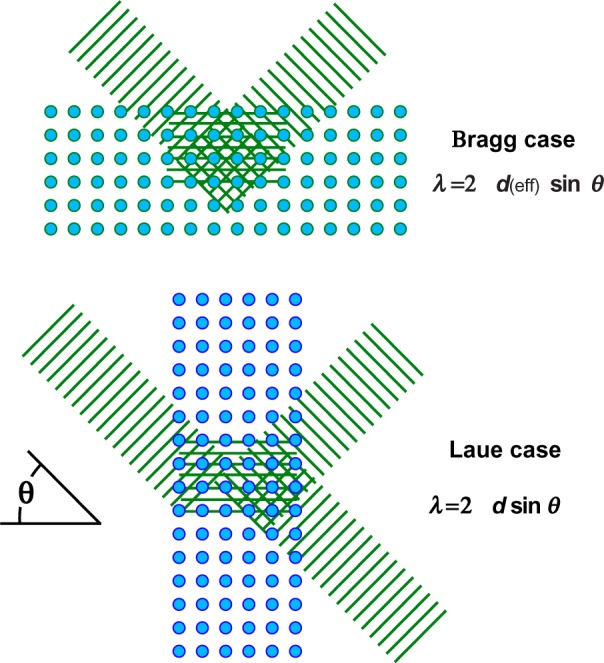
Bragg- and Laue-case reflection conditions.

When the discovery of x-ray diffraction by Friedrich, Knipping, and von Laue [15,16] took place in 1912, the conditions leading to intense diffraction were already at hand both from the work on crossed gratings by von Laue and, as seen later, they were already formulated in Ewald’s thesis [17]. The expressions appeared at first complex, but a useful simplification was shortly introduced by W. L. Bragg [18]. In this view, one considers the diffraction process as arising from interference of waves scattered by sheets of atoms separated by a lattice spacing, *d*, leading to the diffraction condition: λ = 2 *d* sin*θ* for radiation with a wavelength λ. The two geometries in which this condition can be realized are shown in Fig. 1; diffraction in the direction indicated in Fig. 1a is customarily referred to as Bragg-case diffraction, while the situation shown in Fig. 1b is labeled Laue-case diffraction. In a remarkably short time after the initiation of systematic x-ray spectroscopy by Moseley [19,20], the beginnings of γ-ray spectroscopy were already at hand in the work of Rutherford and Andrade [1] as described below. For both wavelength regions, there are certain generalities that need to be considered. The Bragg geometry, which is more suited to longer wavelength spectroscopy, requires that the diffraction condition be modified to allow for the effect of index of refraction. In contrast, symmetric Laue case diffraction requires no index of refraction correction because the effects of directional deviation and wavelength change nicely cancel, allowing the diffraction to proceed as though the scatterers were arrayed in a vacuum.

As it happened, although the earliest crystal diffraction spectroscopy was based on Laue-case geometry, the most impressive early results reported came from adaptation of Bragg-case arrangements to the very small diffraction angles, as will be seen below. As already indicated, crystal diffraction spectrometers directly measure diffraction angles that depend on ratios wavelengths to lattice periods; the results are accurately connected to the base units when the crystal spacing is known in terms of the base unit of length and diffraction angles determined on an absolute scale. In other cases, the spectrometers are primarily interpolators requiring a set of reference lines otherwise established. For some purposes transition energies are more convenient to use than wavelength values. In most cases however, the conversion factor, *hc* = 1.239 842 44(37) MeV·pm is currently sufficiently well established that the translation can proceed in either direction with little degradation in accuracy. In special circumstances, for example for measurements leading to values for the fundamental physical constants, the primary wavelength data must be used directly.

### 2.2 Where It All Began: Rutherford and Andrade (1914)

**Fig. 2:**
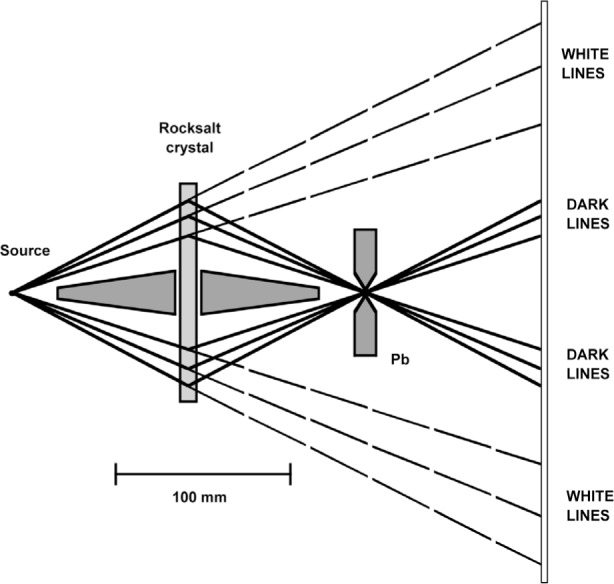
Diagram of the first reported γ-ray spectrograph as described in Ref. 1.

**Fig. 3:**
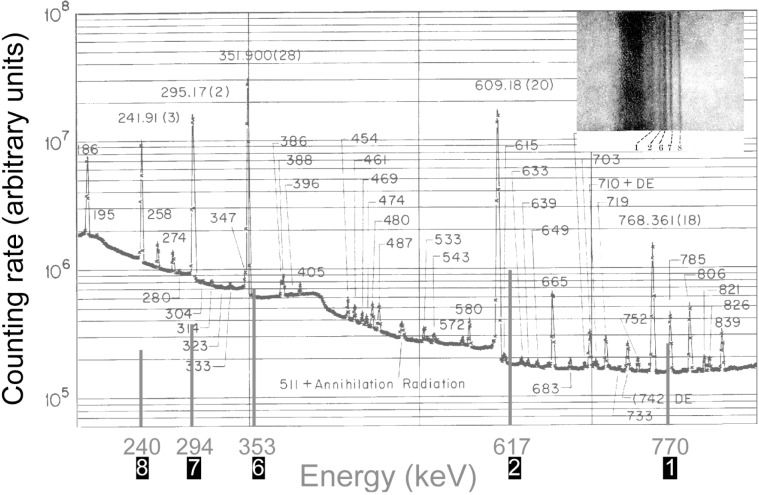
Overlay of modern pulse height distribution with a crystal diffraction spectrum from 1929 [21].

**Fig. 4:**
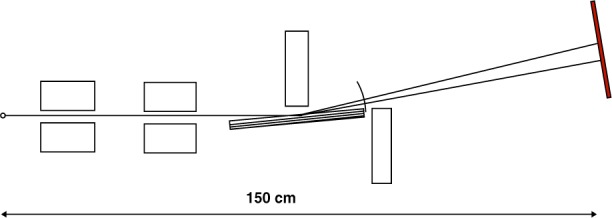
Geometry of the instrument used to obtain the spectrum shown above.

Figure 2 is a diagram of the elegantly simple arrangement employed in what is clearly the first report of γ-ray spectroscopy [1]. The spectra studied included x rays from radium decay products, and γ rays from Radium A and B. From the geometrical parameters (note the compact scale of the instrument and the symmetrically redundant measurement loops), I estimate a potential resolving power of less than 100 even for the longest wavelengths studied. The plates reproduced in Ref. [1] do not have clear images, however, a somewhat later version of a simple flat crystal (Bragg case) instrument described by Frilley [21] yielded spectra that can be put into correspondence with modern ionization spectrometer data, as shown in Fig. 3. Figure 4 is a diagram of Frilley’s spectrograph in which its much larger scale is indicated. This instrument had several interesting features including continuous crystal rotation (to suppress the effects of crystal imperfection), and the use of a calcium tungstate screen to intensify the photographic image. Typical exposures were up to 2 d, during which the crystal (rocksalt, *d* = 2.8 Å) was slowly scanned through an angular range from 0.73° (770 keV) to 3° (185 keV). The larger scale of this instrument suggests an improved resolving power to a value, certainly larger than 100.

### 2.3 Cauchois, Dissertation (1934)

**Fig. 5:**
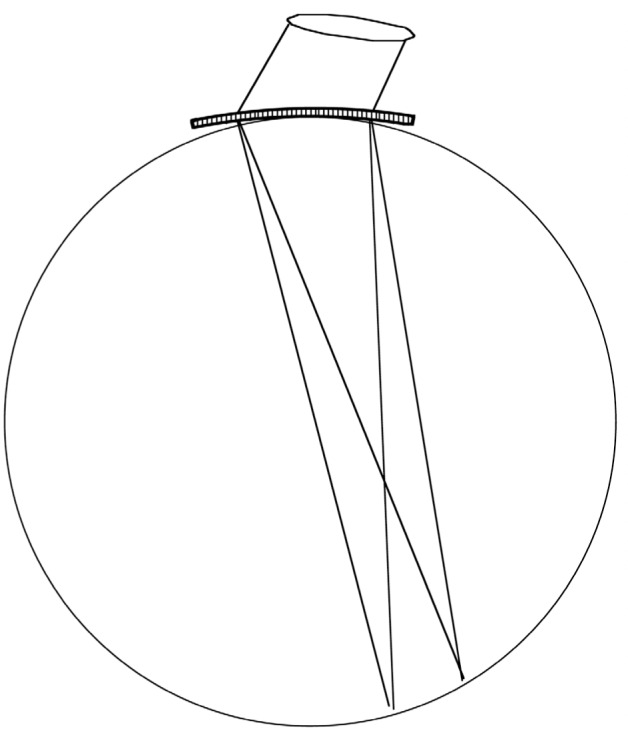
Curved crystal transmission geometry after Cauchois.

The first curved crystal spectrographs, as produced by Yvette Cauchois beginning around 1932, used mica and gypsum crystals and were applied only to x-ray spectroscopy [3–5]. The optical arrangement of what is appropriately referred to as the Cauchois geometry is shown in Fig. 5. When used for x-ray spectroscopy, these instruments are often rather small with focal-circle diameters from 20 cm to 100 cm. Beginning in the 1950s, much larger Cauchois geometry instruments with crystal radii up to 6 m [6] were systematically applied to γ-ray spectroscopy from reactor, accelerator, and radioactive sources. This instrumental geometry is particularly well suited to diffuse sources such as are encountered in accelerator produced sources.

### 2.4 DuMond’s Geometry (1947)

**Fig. 6:**
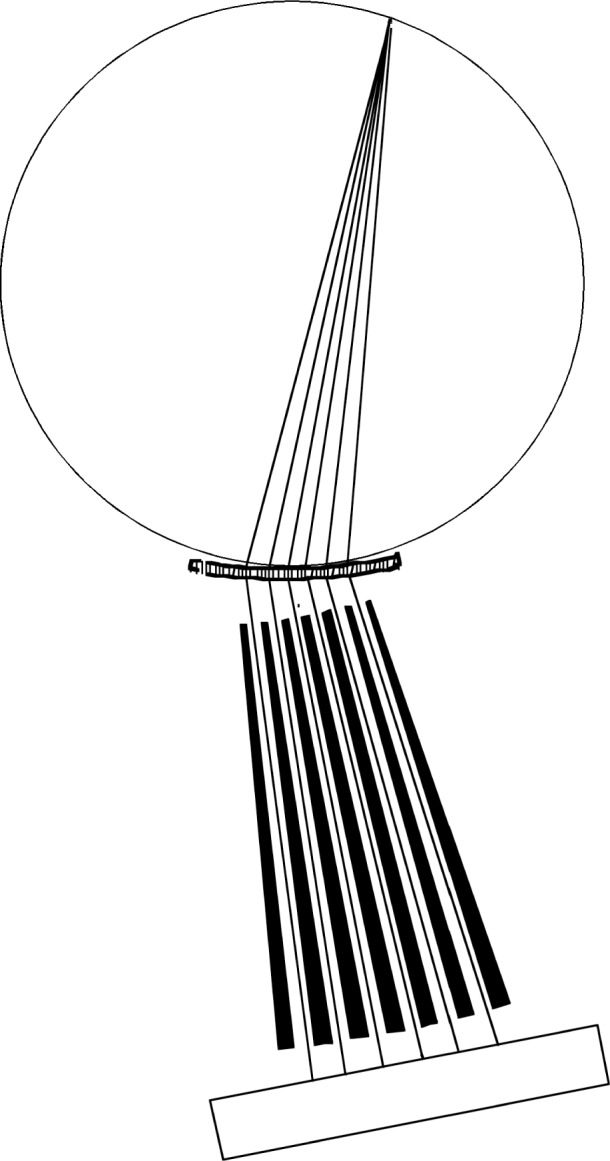
DuMond’s alternative curved crystal geometry [7].

The second alternative for a curved crystal geometry, introduced by Jesse DuMond in 1947, is shown in Fig. 6 [7]. In this arrangement, the resolving power is controlled by ratio of source size to crystal radius, the tapered Soller collimator indicated has no direct role in establishing resolution, its purpose being to shield the necessarily large detector from exposure to direct radiation from the source. Although the initial publication gave only x-ray results, the instrument was intended from the start for γ-ray applications. Indeed, as will be seen below, a considerable number of such instruments have been built and used for γ-ray spectroscopy. The diameters of their focal circles have ranged from 2 m to 24 m as will be seen below. In spite of the stringent requirements on source size, instruments based on this geometry have been by far the most widely applied to γ-ray spectroscopy.

## 3. High Energy, Transmission Geometry Double Flat Crystal Instruments

**Fig. 7:**
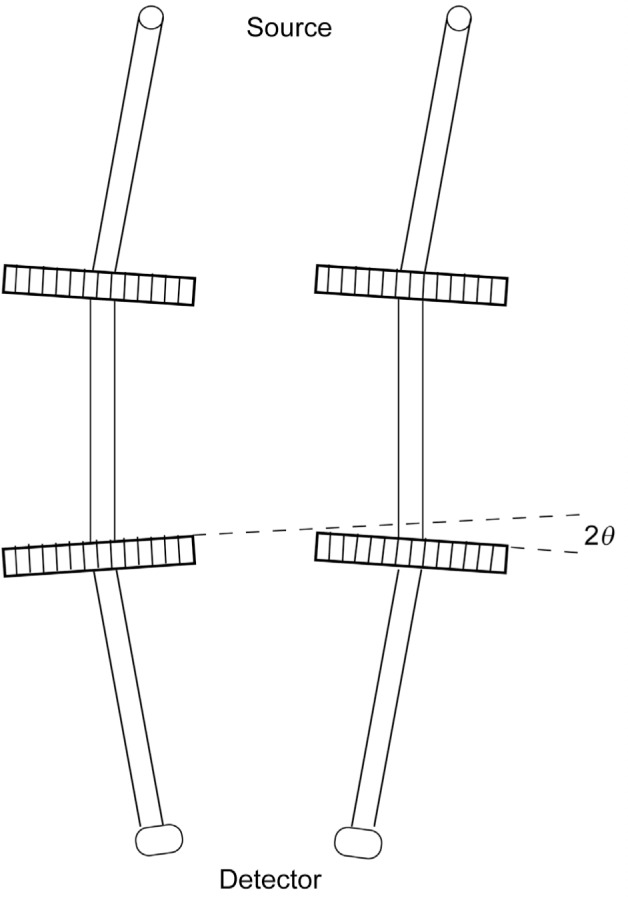
Functional geometry of a two-crystal transmission spectrometer.

Although the first two-crystal instrument that might have been applied to γ-ray spectroscopy was built rather early [12], such application did not occur until very much later [8]. A functional diagram of such an instrument is shown in Fig. 7. As can be seen, there are limitations to the use of such geometry at higher energies (smaller wavelengths). At some point, the nondispersive configuration (oppositely directed diffraction vectors) becomes unusable. Only a very few of these instruments have been commissioned and systematically applied to γ-ray spectroscopy, nonetheless, one recent implementation, GAMS4, has contributed significantly to the work discussed in these proceedings.

### 3.1 State of the Art Diffraction Instruments: 1954–1999

In this section, I propose to follow the paths indicated in the previous section indicating the highest performance implementations of each configuration up to the present time. For the sake of brevity, and at the risk of over-simplification and omission, properties of the three instrument classes identified above have been put in tabular arrays. Entries are not intended to be comprehensive but only representative of the many efforts not indicated in the tables that follow. Table 1 contains descriptions and performance characteristics for a few Cauchois geometry instruments. The following table, Table 2, attempts to provide the same kinds of information for a group of DuMond geometry systems, while Table 3 addresses the same points for the more limited array of double flat crystal instruments. Although the main performance metrics are efficiency and resolution, these numbers cannot be fully appreciated without considering the other constraints on their operation, as can be seen in the cited papers. One clear observation is that the majority of curved crystal spectrometers used in γ-ray applications have been configured in the DuMond geometry, and that the highest performance realization was in the GAMS2, 3 installation at the ILL. In the listing of flat crystal installations in Table 3 the characteristics of GAMS4, as discussed further in the following sub-section, differ radically from the earlier instruments. Differences include the use of thin (highly perfect) crystals, interferometric angle control with absolute calibration, and the achievement of resolving powers greatly in excess of those realized in earlier instrumentation.

### 3.2 High Performance Spectroscopy With GAMS4

**Fig. 8:**
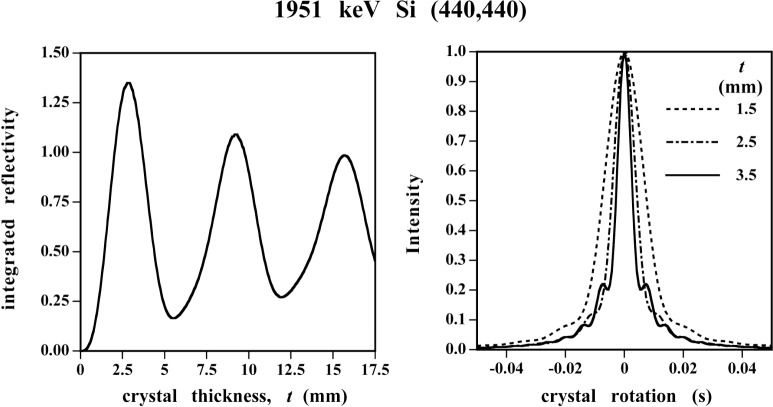
Dynamical diffraction at 2 MeV. Left segment shows integrated reflectivity versus crystal thickness, while the right frame follows diffraction profile changes.

The high resolving power results indicated in the last line of Table 3, and in the report by Kessler and Dewey elsewhere in these proceedings, could not have been realized before the recent availability of highly perfect crystals of silicon and germanium. GAMS4 and its predecessor, NBS-1, were specifically developed to have the ability to exploit such perfection, and to make accurate wavelength measurements well connected to the base units of the SI and fundamental constants such as the Rydberg. In the beginning, there was no assurance that the perfection would be realized. Nevertheless, the instrumentation was designed to fully utilize crystals of extraordinary perfection. Some of the attraction of such an approach can be seen in the simulations given in Fig. 8, where Fig. 8a shows the dependence of integrated intensity on crystal thickness with the two crystals taken to be of equal thickness. Note that for well-chosen values, there is a considerable advantage to be gained. At the same time, as indicated in Fig. 8b, there is an effective narrowing of the spectral pass-band as reflected in the widths of the diffraction patterns shown. Another feature of the diffraction patterns in Fig. 8b is the numerical scale of the abscissa. If such patterns are to be mapped experimentally, then measurement and control are required to be effective at the nanoradian level, i.e., below 0.001”. For accurate wavelength measurements, the angle scale needs to be calibrated *in situ* to this level of accuracy or to a relative refinement of 10^−7^. These requirements have been met through the development of angle measuring schemes based on Michelson interferometry together with the inclusion of indexable optical polygons to enable *in situ* self calibration.

## 4. Conclusion and Outlook

**Fig. 9:**
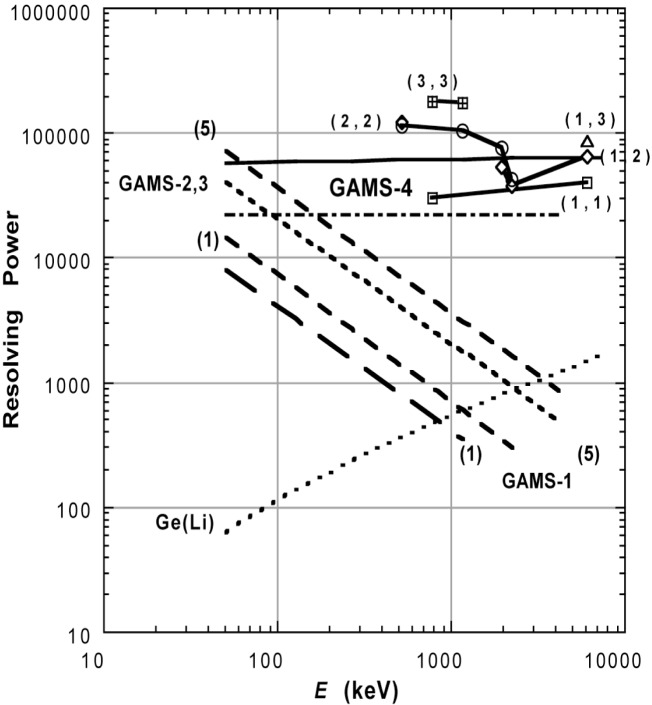
A global view of spectrometer performance as indicated by the single parameter of resolving power.

At the risk of significant oversimplification, I conclude with an overview of several high-performance spectrometer configurations considering only the resolving power parameter. This picture, shown in Fig. 9 has a number of limitations but gives a generally correct overview. One of the limitations is due to the change in lineshape discussed above which makes the uncritical use of pattern widths that vary in strange ways with respect to photon energy. Nonetheless, it is these effective resolving power values that indicate what is of interest, namely how well a sharp line can be located, or how well a complex peakshape can be delineated. Another view of the present and near term accessible performance parameters is indicated in Table 4. Most of the projected improvement, particularly in angle metrology, is based on techniques for reducing periodic error in Michelson interferometers currently under development at NIST [28].

Beyond GAMS4 lies GAMS5 as reported in these proceedings by C. Doll [11]. The main goal of this instrument development is introduction of curved crystals into the two-crystal transmission geometry of GAMS4. This has the potential to dramatically increase the angular acceptance of this instrument in comparison with GAMS4 where the angular acceptance in the plane of dispersion is given directly by the diffraction pattern width. On the other hand, the task of introducing sufficiently uniform large radius curvature in the two crystals and securing the needed alignment is formidable and likely to lead to a limiting value for angular resolution. However, the vastly increased efficiency makes higher order diffraction more accessible, thereby restoring spectroscopic resolution to some extent. My sense is that artful compromise of these issues will lead GAMS5 to a very useful region of performance exceeding the resolving power values realized with GAMS2, 3 and greatly exceeding the efficiencies realized with GAMS4.

## Figures and Tables

**Table 1:** Characteristics of representative Cauchois-geometry instruments

Instrument [Reference]	Radius (m)	Resolving power	Main crystal	Indicator of efficiency
MIT [6,22]	6	870 at 0.1 MeV	Qz (310)	6000 Ci·h (2 MeV)
CIT [23]	2	2000 at 35 keV	Qz (310)	3 × 10^−8^

**Table 2:** Characteristics of some selected DuMond instruments (efficiencies ~ 10^−7^)

Instrument [Reference]	Radius (m)	Resolving power (0.412 MeV)	Minimum width (″)	Main Crystal	Range (MeV)
CIT-Pasadena [7,24]	2	100	20	Qz (310)	0.1 to 2
Argonne [25]	7.7	200	10	Qz (310)	0.1 to 2
KFA-Jülich [13,26]	4.64	500	2.5	Qz (110)	0.03 to 1.5
GAMS2, 3 [9]	24	2000	0.8	Qz (110)	0.1 to 1.5

**Table 3:** List of transmission geometry two-crystal spectrometers for x rays and γ rays

Instrument [Reference]	Inter-axis spacing (m)	Resolving power at (*E* MeV)	Minimum width (″)	Crystal thickness	Range (MeV)
Cornell [12]	1.2	100 at (0.1)	100	Calcite: 10 mm	0.06 to 0.4
Chalk River [8]	1.1	200 at (0.1)	1.8	Calcite: 23 mm	0.1 to 5
Gatchina [27]	0.12	500 at (1.0)	1.0	Calcite: 52 mm	0.5 to 8
ILL GAMS4 [10,14]	0.8	>10^5^ at (1.0)	<0.01	Si or Ge: 2 mm to 4 mm	0.1 to 7

**Table 4:** Current and future performance metrics for two-crystal instruments

Performance metrics	1998	2001
Instrumental linewidth (″)	0.002 to 0.005	0.0005 to 0.0008
Resolving power at 1 MeV	>100 000	400 000
Measurement accuracy	2 × 10^−7^	4 × 10^−8^
High energy limit (MeV)	7	10
